# Aboriginal Women Learning on Country: Lessons for Educators

**DOI:** 10.3389/fpubh.2022.786434

**Published:** 2022-03-31

**Authors:** Lynette R. Goldberg, Dianne Baldock, Terrance Cox, Ha Hoang, Merylin Cross, Andrea D. Price

**Affiliations:** ^1^Wicking Dementia Research and Education Centre, College of Health and Medicine, University of Tasmania, Hobart, TAS, Australia; ^2^Circular Head Aboriginal Corporation, Smithton, TAS, Australia; ^3^Centre for Rural Health, University of Tasmania, Launceston, TAS, Australia

**Keywords:** dementia, Indigenous aging, Indigenous students and employability assets, online undergraduate learning, vocational qualification certification

## Abstract

**Introduction:**

This paper details the journey of eight Aboriginal women from Circular Head, a rural and remote area of North-West Tasmania, as they undertook an innovative 2-year program of tertiary studies in dementia to address a documented community need. The Chief Executive Officer of the Circular Head Aboriginal Corporation (CHAC) had identified difficulties being experienced by older members of the community. These difficulties included changes in behavior, memory, and communication, with profound consequences on social engagement and care needs from both individual and community perspectives. The community wished to know if a combined vocational and university program, completed on Country and in community, could serve as a culturally safe education pathway to empower Aboriginal members of a rural and remote area in providing community health and dementia education and care.

**Methods:**

The nationally funded program included a year-long face-to-face vocational Certificate III in Individual Support (Aging, Home, and Community) on Country, including within-community experience with adults with dementia. This face-to-face learning was combined with online study in the award-winning Bachelor of Dementia care offered by the University of Tasmania. Students received a PhD level stipend to support them in their studies and were guided by an Elder from their community.

**Results:**

All students completed their Certificate III. The number of units they completed toward the eight required for their Diploma of Dementia Care varied. Emergent themes from students' reflections were holistic and relational, highlighting achievements and challenges, the importance of on Country individual connections and community support, and the value of their current and future contributions to the community. Data from this mixed methods approach documented the impact of the innovative coupling of authentic, culturally appropriate experiential learning with broad and deep academic knowledge about dementia and evidence-based care.

**Conclusions:**

This program provided students with a work-related qualification embedded within a university education and increased the capacity and capability of this Aboriginal community to provide care for its members with dementia, a documented concern. The combination of vocational learning on Country with online university study established a pathway to improve students' access to and success in higher education and the professional workforce. This assisted in counteracting the negative influences of racism, stigma, rurality, and socio-economic marginalization on educational opportunity for Aboriginal people. Data showed the need for flexibility with this learning journey, and the strengths and resilience of these women as they learned.

## Introduction

The participation and success of Aboriginal and Torres Strait Islander students in higher education has been hindered by multiple obstacles. In 2008, Andersen, Bunda, and Walter documented lack of physical access; cultural isolation and alienation on campus; dissatisfaction with study options and delivery; inflexibility of higher education systems; unfamiliarity with and lack of confidence in academic requirements and skills; lack of access to educational resources; family and personal disruptions and commitments; and financial issues. These influential writers argued that Indigenous students' success in higher education and subsequent career-based employment needed to be viewed as core university business with collaboration across university programs carefully scaffolded support, and innovative bridging programs as pathways to university study. This core business needed to be flexible and responsive to students' needs with a university-wide understanding of the social, economic, political, and cultural factors that create barriers for Indigenous students, particularly those from rural and lower socio-economic areas.

Subsequent studies (1, 2) expressed concern about continuing inconsistencies and complexities in data collection regarding Indigenous students' success, particularly with needed longitudinal and cross-sectional data to understand the barriers and facilitators students experienced as they moved into university study. Disparities between Indigenous and non-Indigenous students regarding retention and graduation remained a concern, with Indigenous students more likely than non-Indigenous students to leave university without completing their programs of study (3). Following the publication of the Behrendt Review, a special issue of *Learning Communities* (4) highlighted emerging evidence for successful strategies to support Indigenous students in higher education. These strategies included community-wide engagement, smooth transitions in pathways from vocational training to study at university, and access to online education (5).

In 2017, a pivotal partnership between the National Aboriginal and Torres Strait Islander Higher Education Consortium (NATSIHEC) and Universities Australia, the peak body for Australian universities, resulted in the development of an aspirational *Indigenous Strategy 2017–2020* (6) to address the continuing under-representation of Indigenous students. A particular concern was to grow and support the Aboriginal and Torres Strait Islander health workforce with a focus on Indigenous aging (7). Key objectives in this *Indigenous Strategy* included increased enrolment, retention, and program completion by Indigenous students, supported by welcoming and flexible policies and programs. These were complemented by a focus on increasing the understanding and engagement of non-Indigenous staff with Indigenous knowledges, culture, and educational approaches. Further objectives were to recognize, understand, and implement Indigenous research strategies and to take a community leadership role in building opportunities for Indigenous students to attend and succeed at university. These key strategies were incorporated in the University of Tasmania's *Strategic Plan for Aboriginal Engagement 2017–2020* (8).

In a recent special edition devoted to Indigenous issues, Uink et al. (9) detailed the experiences of eight Aboriginal women at different stages in their university studies and urged a focus on the strengths Aboriginal women bring when studying at university, emphasizing how these strengths can facilitate resilience and counteract the challenges they encounter. As more Aboriginal women than men are now entering and graduating from university, recognizing their unique leadership and the ways of being, knowing, and doing they contribute through their lived experiences, memories, relation to Country, and community responsibilities is an important area of discursive enquiry (9, 10). Adding the perspectives of Indigenous women as students to those of Indigenous women as scholars enriches Indigenous Women's Standpoint Theory in its move away from the standpoint of a gender-neutral universal Indigenous experience (9, 10).

The special contribution of Indigenous women became our focus when we were approached by the Chief Executive Officer of the Circular Head Aboriginal Corporation (CHAC) to discuss her concerns about difficulties being experienced by older members of the community. These difficulties included changes in behavior, memory, and communication, with profound consequences on social engagement and care needs from both individual and community perspectives. These difficulties are characteristic of dementia and Aboriginal and Torres Strait Islander peoples are at 3–5 times greater risk (11). The effects of colonization, dispossession, and historical trauma over generations are reflected in documented health disparities between Indigenous and non-Indigenous peoples. Inequities in housing, education, employment, and healthcare are the root causes of a greater burden of chronic disease for Indigenous peoples, including the progressive neurological condition of dementia (12).

The CHAC Board, along with the second and third authors (DB, TC) who are members of the Circular Head Aboriginal community, arranged a series of community yarning sessions to listen, understand, and document community concerns about apparent dementia-related issues. Yarning, a cultural form of conversation to share stories, develop knowledge, and build respectful relationships, is a valid and appropriate qualitative research approach with Indigenous communities (12–14). From these yarning sessions, a major community need was to have emerging leaders of the community educated about and qualified in dementia care so they could then help others understand this progressive condition and address care and risk reduction in culturally appropriate ways (15, 16). A further need was to have the learning occur on Country. “Country” is an holistic concept that encapsulates social and ancestral connections to place. Along with physical, cultural, and spiritual dimensions, it is central to cultural identity. Being “on Country” is restorative, empowering, and healing (17). These important community yarns directly informed our project design of offering the combination of a Technical and Further Education (TAFE) Certificate III qualification in Individual Support (Aging, Home, and Community) and online university study. We were successful in obtaining a 2-year Research and Innovation grant (2017–2019) from the Australian Department of Health/Dementia and Aged Care Services to implement this community-directed plan. This paper details the journey of the cohort of Aboriginal students as they undertook a 2-year program of tertiary studies in dementia, their achievements, and lessons for educators.

### Research Question

Can a face-to-face vocational and online university program, completed on Country and in community, serve as a culturally safe education pathway to empower Aboriginal women from a rural and remote area in providing community health and dementia education and care?

## Methods

### Research Design, Data Collection, and Analysis

This was a single-case, mixed-methods study with a unique “on Country/in community” design. Quantitative data were obtained from students' grades in completing TAFE and university units and their completion of the *Dementia Knowledge Assessment Scale* (DKAS; (18)), measured by *t*-test, at the beginning and end of the project. Qualitative data were obtained from a thematic analysis of students' de-identified reflections from recordings and notes at the beginning and end of the project.

### Recruitment

#### Students

Announcements about the project were posted at the CHAC community office, health center, and on community Facebook pages. The second author (DB), a recognized leader and Elder in the community, spread the word through the community's kinship network. Interested students subsequently explained the project to friends. DB was instrumental in recruiting students for the project. She maintained regular contact with all students, individually, in groups, and via social media throughout the project.

#### A Five-Member Advisory Board

The CHAC Board, which included a second, elected Elder of the community, agreed to serve as the project's Advisory Board. CHAC is an Aboriginal Community-Controlled Health Organisation (ACCHO), offering government-funded home and community support services with specialist services provided by visiting non-Aboriginal health professionals. There are ~1,100 Aboriginal people in the Circular Head community, with 445 aged 35 years and over. There is a 61-bed residential aged care center in the general community.

#### Project Officer

A detailed position description was developed and posted within the Circular Head community, specifying the responsibilities of the full-time position and the need for it to be filled by an Aboriginal person familiar with the community. The three Aboriginal applicants were interviewed by Aboriginal and non-Aboriginal members of the project team and members of the CHAC Board. Grant funding specified the salary for the Project Officer. However, university policy stipulated that, for such a salary, the person in this position needed certain qualifications which our preferred candidate did not have. This negotiation delayed her employment and provided insight into how the Western structure of university policies, as well as curricular content, needs to change to reflect the cultural diversity and contribution of Aboriginal communities in Australia.

### Establishing the Program

#### Re-locating the Certificate III Program

This program was typically offered in Burnie, an hour's drive away from the community. The Division Manager for Human Health and Business Services at TAFE Tasmania (TasTAFE) was contacted, informed about the grant, and asked if it was possible to deliver the Certificate III program in the community for a year. He agreed, and TasTAFE was compensated by grant funding for the instructor's costs in traveling to provide face-to-face instruction for 1–2 days each week during the first academic year of the project.

#### The Bachelor of Dementia Care

This is a fully online program open to all but specifically designed to support mature-age learners and those who may not have completed Grade 12. There is no required Australian Tertiary Admissions Rank (ATAR) score. In addition to teaching staff who coordinate the units, the program has two full-time academic support staff to assist students with enrolment, assignments, timelines, and general study skills. The first eight units in the program are tuition-free, supported by the Australian Government's Higher Education Participation Program (HEPP) initiative and a waiver of the Higher Education Contribution Scheme (HECS). These eight units comprise a Diploma in Dementia Care. Three of the early units include tertiary preparation content. Continuing students can graduate with an Associate degree in Dementia Care after completing 16 units, or with the Bachelor degree after completing 24 units. In 2016, the program received an Australian Government, Department of Education and Training, University Teaching Award for programs that enhance learning and widening participation in dementia education (19).

### Procedures

The 2-year grant commenced in July 2017. Ethics approval, recruitment, and program organization took place from July to December in preparation for students to commence their face-to-face TAFE and online BDC studies the following year. Community meetings were held in September, October, and November for interested members and students to talk about the project, and for the project team to update the CHAC Board. During this time, the project team and an Aboriginal Teaching and Learning consultant planned to review the first 16 units of the university program to ensure the content was culturally safe. However, following the consultant's suggestion, this plan changed to focus on program staff and engage them in a series of activities to learn about the history of Aboriginal people in Tasmania, their nine nations and multiple clans, their cultures, knowledges, history of colonization and dispossession, and the ongoing intergenerational disparities in health, education, and employment.

Students commenced their face-to-face TAFE and online university studies in Semester 1, 2018. The TAFE program concluded in December 2018. Online university studies supported by the grant continued through October 2019. Throughout the project, the full-time female Aboriginal Project Officer facilitated learning in weekly group meetings and visited students at their homes. In Year 1, students also had access to Aboriginal student support personnel at TasTAFE. Throughout the project, students were encouraged to maintain contact with staff at Riawunna, the university's Aboriginal Student Centre.

During Semester 1, 2018, TAFE classes were held at a vocational education and training center on Mondays. In Semester 2, additional classes were held on Tuesdays to prepare students for their upcoming hands-on experiences in the community and in residential care. The plan was for students to use the remaining three work-week days in 2018 for their online university studies, and to have the full week available for university study in 2019.

The CHAC/Advisory Board approved the use of the *Dementia Knowledge Assessment Scale* (DKAS; (18)) to document students' understanding of dementia at the beginning and end of the project. Students were given dictaphones and encouraged to record, write down, or share reflections and insights verbally with their Project Officer, the second author (DB, an Elder in the community) and other members of the team as the project continued.

The project was approved by the university's Social Sciences Ethics Committee, H0016737. This included adherence to *Ethical Conduct in Research with Aboriginal and Torres Strait Islander Peoples and Communities* (20).

## Results

Twelve students were initially recruited for the project's 10 funded positions. The first 10 applicants were funded. Each funded student received a doctoral-level stipend for each year of the project. All students were local Aboriginal women, and all (funded and unfunded) commenced the program in Semester 1, 2018. Four of the funded students left early in the first semester. One decided that a focus on aging and dementia was not for her; one moved into Midwifery studies, and two were not able to continue due to serious personal circumstances. Their leaving enabled the two “reserve” students to be funded. The eight women in the project cohort were aged from 18 to 49 years. Two students had completed Year 10, four had completed Year 11, and two had completed Year 12. Between them, prior to starting the project, they had received the following qualifications: Certificate II in Business (*n* = 1), Certificate III in Community Services (*n* = 1), Certificate IV in Community Services (*n* = 2), Diploma of Agriculture (*n* = 1), and a Diploma of Management (*n* = 1). Two more students were recruited as Semester 1 progressed but did not continue. The savings from these two un-filled funded positions enabled the project to continue for an additional semester, providing five semesters of university study.

The initial plan for concurrent face-to-face TAFE and online university study is detailed in Table 1. The intended outcome was students' completion of the TAFE Certificate III in Individual Support (CHC33015) at the end of Year 1 and completion of the Diploma of Dementia Care (eight units) at the end of Year 2. Students were asked to complete Units 1 and 2 in the Diploma course first as these were tertiary preparation units. The order in which they completed the remaining six Diploma units could then vary.

**Table 1:** Outline of the initial 2-year plan of study.

	**Semester 1**	**Semester 2**	**Spring school extended (optional university study)**
**Year 1**	TAFE^*^	TAFE	TAFE
	BDC^**^Unit 1	BDC Unit 3	BDC Unit 2
	BDC Unit 2	BDC Unit 4	BDC Unit 4
**Year 2**	BDC Unit 5	BDC Unit 7	
	BDC Unit 6	BDC Unit 8	

* *The TAFE CHC33015 Certificate III units are detailed at http://www.training.gov.au/*.

** *BDC, Bachelor of Dementia Care (online). Unit 1: CAD001-Learning Online in Health Studies; Unit 2: CAD002-Introduction to Effective Communication in Health Studies; Unit 3: CAD003-Introduction to General Studies in Dementia in Australia; Unit 4: Neurospeak-Understanding the Nervous System; Unit 5: CAD101-Introduction to Aging, the Brain and Dementia; Unit 6: CAD102-Introduction to Population Trends in Aging; Unit 7: CAD103-Introduction to Dementia Services; Unit 8: CAD104-Principles of Supportive Care for People with Dementia*.

### Students' Grades

All students successfully completed the TAFE Certificate III in Individual Support (Aging, Home, and Community) in Year 1. This qualification equipped them with work-ready skills to provide person-centered support to people in need in the community and residential care who were aging and/or with a disability. The qualification entailed successful completion of seven core and six elective units, along with a minimum of 120 h of practical experience which was undertaken in-community. The students also completed a First Aid elective. Assessment tasks in each unit measured competence (pass/fail) through written and oral questions, case studies, and demonstration of ability during practical tasks, simulations, and on-the-job responsibilities in home, community, and residential care placements.

The TAFE course was taught by an experienced Registered Nurse who was not Aboriginal. The Aboriginal Project Officer sat in on the classes. On occasion, the Project Officer and TAFE instructor needed to discuss and reconcile disagreements about instructions and approaches to assessments. Students found the instructor's authoritarian approach difficult at times but understood her desire to help them learn, and persevered. The constructive discussions between the Project Officer and TAFE instructor provided a guide for students as they moved into online university studies and needed to pose questions on Discussion Boards.

For most students, studying TAFE and university units at the same time proved difficult and so the plan of university study became more flexible. Over the 2-year grant period, the number of units students were able to complete toward the eight required for the Diploma of Dementia Care ranged from 1 to 5 (Table 2).

**Table 2:** Students' progress and grades^*^ in university units through the five grant-funded semesters; TAFE studies were completed in Year 1.

**Student**	**Semester 1, 2018**	**Semester 2, 2018**	**Spring school extended 2018–2019**	**Semester 1, 2019**	**Semester 2, 2019**
1	TAFE √^*^	TAFE √	TAFE √	Unit 5—Withdrew due to	Unit 6—Withdrew
	Unit 1—P ^**^	Unit 2—DN	Unit 4—CR	family commitments	
2	TAFE √	TAFE √	TAFE √	Unit 5—F	Withdrew due to family
	Unit 1—DN	Unit 2—DN	Unit 4—CR		commitments
3	TAFE √	TAFE √	TAFE √	Unit 5—P	Unit 6—P (continued studies
	Unit 1—HD	Unit 2—HD	Unit 4—DN		after the grant ended)
4	TAFE √	TAFE √	TAFE √	Unit 4—Withdrew due to	Unit 5—P
	Withdrew from BDC to focus on TAFE studies	Unit 1—P	Unit 2—P	family commitments	
5	TAFE √	TAFE √	TAFE √	Unit 4—Withdrew due to	
	Withdrew from BDC to focus on TAFE studies	Unit 1—P	Unit 2 -Withdrew due to health problems	health problems	
6	TAFE √	TAFE √	TAFE √	Unit 4—F	Unit 4—P
	Withdrew from BDC to focus on TAFE studies	Unit 1—P	Unit 2—P		Unit 5—F
7	TAFE √	TAFE √	TAFE √^***^	Unit 4—Withdrew due to	Unit 4—Withdrew due to
	Withdrew from BDC to focus on TAFE studies	Unit 1—CR	Unit 2—P	health problems	health problems
8	Not yet in program; Previous TAFE qualification	Unit 1—P	Unit 2—CR	Unit 4—P	Unit 5—P (continued studies after the grant ended)

* *√, successful completion of TAFE units*.

** *Grades for BDC units are F, Fail (below 50%); P, Pass (50–59%); CR, Credit (60–69%); DN, Distinction (70–79%); HD, High Distinction (80–100%)*.

*** *Nominated for TasTAFE Indigenous Student of the Year*.

### Pre- and Post-project DKAS Scores

Responses to the 27 items on the DKAS used a 5-point Likert scale: *false, probably false, true, probably true*, and *don't know*. The 27 items addressed the causes, characteristics and types of dementia, risk factors, and care needs. *Correct* responses scored 2 points and *probably correct* responses scored 1 point. Responses that were *incorrect, probably incorrect*, and *don't know* received no points. The maximum achievable score was 54 points. Students' mean pre-project score was 20.12 (range = 4–38; standard deviation = 11.70). Lower scores were characterized by frequent *don't know* responses. Asked to rate their current knowledge of dementia on a 5-point scale (1 = no knowledge; 5 = high knowledge), students rated themselves a 1 (*n* = 3), 2 (*n* = 3) and 3 (*n* = 2). Students' mean post-project score was 42.17 (range = 37–49; standard deviation = 4.44). The positive difference in pre- to post-project scores was statistically significant (*t*_pre_ = 4.86, *df* = 7; *t*_post_ = 23.23, *df* = 5; *p* = 0.002). As measured by correct DKAS scores, there were notable increases in students' understanding of (i) changes in the brain related to dementia; (ii) other forms of dementia, including vascular dementia, for which Aboriginal people are particularly at-risk due to chronic health issues such as cardiovascular problems; (iii) the positive effects of a healthy lifestyle and modifiable risk factors to reduce dementia risk; and (iv) the importance of an early diagnosis. Students' continued to be confused about the use of medications to alleviate changes in behavior associated with dementia—whether medications should be the first choice or whether medications should be prescribed if non-pharmaceutical interventions were not effective. Their confusion was understandable as they had not yet completed university units addressing such complexities in care. Their post-project self-rating of current knowledge of dementia increased: 3 (*n* = 2), 4 (*n* = 6).

### Thematic Analysis of Students' Reflections

Only two students maintained reflective recordings. Others were comfortable in discussing their experiences and having project team members write summary notes but were reluctant to have their feelings and experiences recorded. Transcriptions and verbatim comments were shared with students to ensure accuracy and approval to use.

The Indigenous standpoint guiding this project constitutes the cultural interconnectedness of Aboriginal ontology (ways of being), epistemology (ways of knowing), and axiology (ways of doing) (10). The emergent themes are holistic and relational, rather than discrete categories, and highlight the women's achievements and challenges of studying, the importance of on Country individual connections and community support, and the value that each can bring to their community now and into the future. Students' ways of being, knowing, and doing were evident as they reflected on their experiences. Despite their initial concerns, these women spoke of their TAFE and university experiences in a positive light:

*My experience studying the Certificate III has affected me tremendously. I commenced my journey feeling extremely apprehensive, quite anxious, and somewhat afraid. I have always suffered with low self-esteem and lack confidence. However, on completion of the course I am a new person as I overcame many challenges. I am now proud of who I am and what I have achieved in a short amount of time*.

Several spoke of their personal and professional achievements shaping their new sense of self:

*I have benefited from the university study in many ways. Academically I didn't feel I was there at first - I felt overwhelmed and out of my depth - but I have learned many new skills and gained knowledge. This is helpful on both personal and professional levels*.

They also expressed a new capacity to guide and empower other community members:

*I believe I have turned my life around. My message is “Anything is possible. Allow yourself to be a beginner. No-one starts off being excellent. Follow your heart and never give up on your hopes and dreams.” I would like to pass this on to others so they too could develop their potential*.

One student had a baby while undertaking her studies and spoke of new relational ways of being, knowing, and doing in meeting the challenges of motherhood and study:

*I thought the experience was a wonderful journey. The TAFE was a very big challenge with having a newborn, but I managed to finish strong and loved every minute of it*.

Their studies facilitated a new focus on future career pathways:

*I now know what career path I would like to pursue. Stepping outside of my comfort zone was extremely difficult but I am so grateful I did*.

Students spoke of gaining confidence through their studies that translated into enhanced cultural ways to support other Aboriginal community members:

*I have gained so much from going back to education as a mature-age student. I know what I have to offer the people in my community*.

*I just finished high school and now have a qualification for work in aged care which I love*.

The supportive group learning environment on Country gave students the opportunity to develop confidence and undertake new ways of being in leadership roles:

*I took on a leadership role in my university studies. I was nominated to be the leader in our group assignment. Although it was quite daunting for me at the time, I came away from it very encouraged as the other members in my group gave me positive feedback*.

On Country learning enhanced the resilience of these women with vital community support. They also developed their own social and cultural support network through their studies:

*Supporting each other is the most important thing. As we were all going on the same journey, we made strong connections and established life-long friendships*.

*I have developed vital networks which I turn to when I need assistance or support. Their generosity in time, personal support, and their expert information is nothing short of amazing*.

*Studying in-community was great – having the support of Di and like-minded people we know; not having to travel over an hour away to the closest campus*.

*We couldn't have done this without Di. She never stopped believing we could do it*.

The social and cultural support networks also provided a protective factor for dealing with the challenges of online learning, unreliable internet access, the Western approach to teaching, and the challenges of balancing life and study:

*The internet coverage was difficult at times. The language content in the BDC was also a bit daunting, as well as the expectations from the lecturers and the lack of face-to-face contact*.

*The BDC teaching material needs to be considered to capture the Aboriginal audience in a culturally respectful manner. Language is important. Content could be made simpler. Having a unit dedicated to dementia in Indigenous peoples would be a fantastic idea*.

*The last few weeks have been the most difficult in my entire life. I have found myself in a rut that I didn't know whether I would be able to get out of. The assignments are getting harder every semester and even though I am trying my hardest, I am receiving marks I am not happy with…. I am remaining calm and I am proud of how far I have come, and I will not give up on myself*.

At the end of the 2-year grant period, three students were not working—one due to a new baby, and two to family farming needs. The remaining five students were employed in community, residential, or hospital-based positions due to their TAFE qualification and dementia experience. The students reinforced the value of the program in developing new ontological ways of being Aboriginal students and health workers, new epistemologies in infusing Aboriginal cultural knowledge with Western dementia education, and new ways of providing dementia care while connected to family, community, and Country.

*When I was completing my work placement in an aged care facility, I had comments like, “How could you possibly do a degree in dementia? They do not know enough about it.” In a sense, they were right – people do not know enough about dementia. So, this program we are in will benefit not only families but whole communities – we will have the qualifications to deliver a better standard of care to those diagnosed with dementia*.

The achievements of these women were further supported by employers who commented on their “*remarkable ability to work with older people, especially those with dementia*.” The results strongly reflect the relational and interconnected ways of being, knowing, and doing these Aboriginal women experienced with the achievements and challenges of simultaneously studying TAFE and university subjects while connected to Country and community. These women were asserting their own place in the world, taking some control of their lives, and determining their own futures.

## Discussion

This grant-funded project addressed a community-initiated question: whether a face-to-face vocational and online university program, completed on Country and in community, could serve as a culturally safe education pathway to empower Aboriginal women from a rural and remote area in providing community health and dementia education and care. The effect of the 2-year program on the achievements of a cohort of eight Aboriginal women in a rural and remote community was positive and profound. Organizational support to move the TAFE program to their community coupled with online university study, sustained guidance from a respected community leader, and consistent community support of both younger and mature-age students (21) enabled achievement of long-held individual dreams and addressed community concerns, providing benefits to all. Two women are continuing their studies in the online Bachelor of Dementia Care (one completed Grade 10; one completed Grade 12). One has also been recruited into an online Graduate Diploma in Indigenous Health Promotion at a mainland university. Five women are employed in the community as a result of their TAFE qualification; one was nominated for Indigenous Student of the Year, and one plans to enroll in the newly available Bachelor of Nursing program in Burnie. One program participant continues to farm but celebrates her qualification. All report having found a sense of meaning and purpose from their studies (9).

In recognition of their accomplishments, the women were invited to discuss their experiences at the 2019 Australian Dementia Forum. At this conference, they met and conversed with national Aboriginal leaders in aging, dementia care, and health promotion and wellness. Our granting agency, the Department of Health, highlighted the students' achievements in a national “Good News Story” email entitled *Caring into the Future*.

For Aboriginal students in rural and remote locations, such as Circular Head, this project showed that a community-based vocational to online university education pathway, guided by a community leader (the second author) and accommodating students' personal circumstances, was possible. It increased students' assurance and established a foundation for continued online education in general health promotion and dementia education. This cohort of Aboriginal women faced the barriers well-documented in the literature: living in a rural and remote area with aspects classified as lower socio-economic; the closest university campus being an hour away with (at that time) a limited choice of programs; lack of transport; and multiple family and work responsibilities. Several students were dealing with serious physical and mental health issues (3, 9, 22). Becoming aware of the negative issues experienced by these students brought home to university staff the ongoing consequences of colonization and the multiple inequities experienced by Aboriginal people in Tasmania.

With their quiet strength, and the care and education about dementia they were providing in the community, the students in this project became role models and mentors for high school students interested in health education (2). They participated in writing a successful university grant that was funded through the university's Indigenous Student Success Program. This grant supported their interactions with Aboriginal High School students to interest these students in university study. Five High School students were recruited, enrolled in university programs, and given grant-funded personal computers to assist their studies (23). As their confidence grew, those who continued in the university program shared cultural approaches to care on unit Discussion Boards and in their assignments, linking Western and Aboriginal knowledges and perspectives. This shared knowledge benefitted both non-Indigenous students and teaching staff and provided a small step toward establishing culturally responsive content and curriculum reconciliation in health education (10, 24). The students suggested developing a new unit in which Aboriginal knowledges could be embedded in dementia care. The Jindaola program (24) provides a model for how this could be done. In the meantime, all program staff now authentically Acknowledge Country at the beginning of lectures and meetings.

Consistent access to the Internet was a challenge in this rural and remote community but the Aboriginal community center and the local library provided free access when needed. The onsite support of the Aboriginal Project Officer facilitated the smooth transition from the TAFE program into university study, addressing the strategy recommended by Smith et al. (5) and incorporated in the University of Tasmania's *Strategic Plan for Aboriginal Engagement*. For some students, the contrast between the TAFE face-to-face task-oriented learning and the theory-driven online university learning remained difficult. Students were reluctant to telephone or email the two BDC Student Support staff. In retrospect, organizing videoconferencing contact may have been valuable, so students could match names with faces and decrease their reliance on members of their community. However, community members welcomed opportunities to show their support. Indeed, such community support may strengthen and sustain the needed relationships between Aboriginal communities and universities (6, 24), guiding students in their learning and research and helping all students learn *from* Aboriginal culture (24). Such community support may also facilitate recognition of Aboriginal students' community work experience as a component of their university learning—an issue that warrants further consideration in online programs focused on Western approaches to health care.

One may question whether there is a tension in the pathway from vocational to university qualifications as most of the women in this project, at the time of manuscript submission, were not continuing with university study. Is it a concern that students view a TAFE qualification as “enough” (2)? In response, we would suggest that achieving a TAFE qualification that did not seem possible due to geographic distance and the crushing issues of intergenerational trauma, racism, and stigma causing low self-esteem, as evidenced in students' comments, was an achievement to be celebrated. The achievements of these Aboriginal women challenged gender, race, rurality, and socio-economic expectations. Their achievements were a recognized step in addressing their community's documented concerns about care for members who were aging, at-risk for dementia, or recognized as living with this condition.

To our knowledge, this is the first example of a vocational-online University pathway for qualifications in dementia care. The responsibility to show how such a vocational qualification transfers into and is recognized by a university appears to rest with those of us at universities to help students understand the choices they have for the career pathways they wish to pursue (4, 9). Any university asking a potential student, “*If you would like to do this,”* must recognize its role in understanding and committing to addressing “*How can we help?”* This assistance needs to include active partnerships with Aboriginal communities to address the challenges many students continue to face as they undertake university study (5, 9, 22, 24) and public encouragement and support (21). University assistance also needs to recognize that Aboriginal students' journeys in tertiary education may need to be flexible, with time for reflection and contemplation to make sense of what has been learned and how this relates to one's cultural sense of self. At the beginning of the project, as a component of the university enrolment process, students were asked about their achievements since high school. They initially responded “Nothing.” This was not the case. Whether they were farming, volunteering, or providing care to children and adults, they were involved in strategic planning, organization, and collaborative activities to support their community. This highlighted the importance of encouraging students not to give away their worth but to view their previous accomplishments and cultural framework as relevant to higher education. When students are enrolling at university, the wording of questions related to experiences prior to university study needs to encourage Aboriginal students to realize that their ways of being, knowing, and doing through their lived experiences, community responsibilities, and previous accomplishments are valued by the university (9, 10, 24).

Since the project ended, the university has changed its service delivery model from a hub-and-spoke system to three distinct, place-based campuses offering greater program choice to meet the needs of the surrounding communities. One of these campuses is in the North-West of Tasmania. The university has also created a new part-time BDC position, based in the North-West, to provide direct face-to-face contact and support for students studying online. Ideally, over time, this support can increase to include paid Aboriginal staff in rural and remote communities.

## Conclusion

In line with Universities Australia's (6) *Indigenous Strategy*, this funded 2-year project featured a unique “on Country/in-community” design to support eight Aboriginal women to complete a TAFE Certificate III as a pathway to ongoing university studies in dementia care. This initiative addressed a documented community need. The project also stimulated opportunities for non-Aboriginal university staff to increase their understanding of, and engagement with Aboriginal knowledges, culture, and research strategies. This project was the first time a cohort of Aboriginal students had been recognized and listened to in the BDC program, helping to establish a shared process to ensure culturally responsive content in online dementia education. The achievements of these eight Aboriginal women ripple out. Their successes in higher education and career pathways serve as role models for their families and community members and provide their community with improved capacity to “look after their own” and others into the future.

We acknowledge the Aboriginal community leaders for ensuring the project methodology progressed within this culturally embedded ontological, epistemological, and axiological framework.

## Data Availability Statement

The data presented in the study are deposited in the University of Tasmania open access repository, accession number 149101; available through https://utas.libguides.com/OpenAccess/OAR.

## Ethics Statement

The studies involving human participants were reviewed and approved by the University of Tasmania Social Sciences Ethics Committee, H0016737. This included adherence to Ethical Conduct in Research with Aboriginal and Torres Strait Islander Peoples and Communities (20). The patients/participants provided their written informed consent to participate in this study.

## Author Contributions

LG and DB worked with the students and collected data. LG, DB, and TC drafted the manuscript. MC, HH, and AP contributed revisions. All authors wrote the grant which funded this work and approved the final version of the manuscript.

## Funding

This work was supported by a grant from the Australian Department of Health/Dementia and Aged Care Services (DACS) (grant number: 4-4ZPGDS7).

## Conflict of Interest

DB was employed by the Circular Head Aboriginal Corporation. TC is a member of the Aboriginal Community and not employed by the Circular Head Aboriginal Corporation. The remaining authors declare that the research was conducted in the absence of any commercial or financial relationships that could be construed as a potential conflict of interest.

## Publisher's Note

All claims expressed in this article are solely those of the authors and do not necessarily represent those of their affiliated organizations, or those of the publisher, the editors and the reviewers. Any product that may be evaluated in this article, or claim that may be made by its manufacturer, is not guaranteed or endorsed by the publisher.
